# Optical Redox Imaging of Ex Vivo Hippocampal Tissue Reveals Age-Dependent Alterations in the 5XFAD Mouse Model of Alzheimer’s Disease

**DOI:** 10.3390/metabo12090786

**Published:** 2022-08-25

**Authors:** He N. Xu, Sarah Gourmaud, Allison Podsednik, Xiaofan Li, Huaqing Zhao, Frances E. Jensen, Delia M. Talos, Lin Z. Li

**Affiliations:** 1 Britton Chance Laboratory of Redox Imaging, Department of Radiology, Perelman School of Medicine, University of Pennsylvania, Philadelphia, PA 19104, USA; 2 Department of Neurology, Perelman School of Medicine, University of Pennsylvania, Philadelphia, PA 19104, USA; 3 Center for Biostatistics and Epidemiology, Department of Biomedical Education and Data Science, Temple University Lewis Katz School of Medicine, Philadelphia, PA 19140, USA

**Keywords:** NAD redox, FAD-containing flavoproteins, redox ratio, amyloid-β, redox shift

## Abstract

A substantial decline in nicotinamide adenine dinucleotide (NAD) has been reported in brain tissue homogenates or neurons isolated from Alzheimer’s disease (AD) models. NAD, together with flavin adenine dinucleotide (FAD), critically supports energy metabolism and maintains mitochondrial redox homeostasis. Optical redox imaging (ORI) of the intrinsic fluorescence of reduced NAD (NADH) and oxidized FAD yields cellular redox and metabolic information and provides biomarkers for a variety of pathological conditions. However, its utility in AD has not been characterized at the tissue level. We performed ex vivo ORI of freshly dissected hippocampi from a well-characterized AD mouse model with five familial Alzheimer’s disease mutations (5XFAD) and wild type (WT) control littermates at various ages. We found (1) a significant increase in the redox ratio with age in the hippocampi of both the WT control and the 5XFAD model, with a more prominent redox shift in the AD hippocampi; (2) a higher NADH in the 5XFAD versus WT hippocampi at the pre-symptomatic age of 2 months; and (3) a negative correlation between NADH and Aβ_42_ level, a positive correlation between Fp and Aβ_42_ level, and a positive correlation between redox ratio and Aβ_42_ level in the AD hippocampi. These findings suggest that the ORI can be further optimized to conveniently study the metabolism of freshly dissected brain tissues in animal models and identify early AD biomarkers.

## 1. Introduction

Alzheimer’s disease (AD) is a progressive neurodegenerative disease most often associated with memory deficits and cognitive decline. The distinct neuropathological features identified in AD patients include β amyloid (Aβ) plaques, neurofibrillary tau tangles (NFT), and pronounced neuronal loss, leading to brain atrophy. Furthermore, multiple metabolic abnormalities, including glucose hypometabolism, lipid dysregulation, alterations of the tricarboxylic acid (TCA) cycle, impaired mitochondrial oxidative phosphorylation (OXPHOS) activities, and increased oxidative stress have been identified in AD brains [1,2,3,4,5,6,7,8]. While it is still unclear how these metabolic abnormalities contribute to AD pathology, emerging evidence shows that metabolic alterations precede the appearance of amyloid plaques and cognitive decline [2,6,9,10], suggesting that metabolic alterations may represent an early event in AD progression. 

Nicotinamide adenine dinucleotide (NAD, including the oxidized form NAD^+^ and the reduced form NADH) and flavin adenine dinucleotide (oxidized form FAD and reduced form FADH_2_) are essential co-enzymes for the TCA cycle and the electron transport chain (ETC). FAD is an electron acceptor, similar to NAD^+^ in its action. By binding to proteins, they critically support cell energy metabolism and maintain mitochondrial redox homeostasis. NAD redox sites are critical control sites for metabolic shifts in neurons for aging and AD mouse models [11]. NAD deficiency and the resulting alteration of the NAD redox status have been previously associated with aging and neurodegenerative declines in culture models [9,11,12,13,14]. A more oxidized redox status (i.e., a higher FAD/NADH ratio) has been observed in cultured hippocampal and cortical neurons isolated from 4 month 3xtg-AD mouse brains, prior to increased reaction oxygen species (ROS) and oxidative stress that promotes neurodegeneration at 10 months of age [9,15]. In AD mouse models, brain NAD^+^ deficiency was associated with compromised cellular bioenergetics, which may contribute to AD pathogenesis, as supplementation with NAD precursors at a young age mitigates neuropathology and/or neurobehavior deficits [16,17,18,19,20,21]. Similarly, a deficiency in brain NAD^+^ levels was implicated in the symptomatic stage of AD patients [13]. However, the NAD redox status during AD progression has not been systematically characterized at the tissue level. Both NADH and FAD are intrinsically fluorescent, and their fluorescent intensities indicate the physiological status of cells [22]. Hence, alterations in NADH and/or FAD contents may indicate pathological conditions. 

The optical redox imaging (ORI) technique measures the intrinsic fluorescence intensities of cellular NADH and FAD-containing flavoproteins (Fp). The redox ratio Fp/(NADH+Fp) is sensitive to mitochondrial redox status, cellular metabolism, and oxidative stress [22,23,24,25,26,27,28,29]. Here, we report a new application of a convenient and quick quantitative ORI technique to image freshly dissected mouse hippocampal sections ex vivo. By using a wide-field fluorescence microscope to acquire NADH and Fp intensities, we characterized the redox status of the hippocampus of a well-characterized AD mouse model with five familial Alzheimer’s disease mutations (5XFAD) and wild type (WT) control littermates at various ages spanning the full range of the course of the disease.

## 2. Materials and Methods

### 2.1. Animals and Brain Tissue Dissection

#### 2.1.1. Mice

A total of 21 heterozygous 5XFAD (Tg6799 line) mice (7 male, 14 female) and 21 wildtype (WT) littermates (7 male, 14 female) were used for this study (Supplemental Tables S1 and S2). Mice were originally obtained from the Jackson Laboratory (MMRRC stock #34840, Bar Harbor, ME, USA) and maintained on a mixed B6SJL genetic background. The 5XFAD transgenic mice carried five mutations seen in patients with the genetic familial form of the disease: the human APP gene with the Swedish K670N/M671L, Florida I716V, and London V717I mutations, and the human PSEN1 gene with M146L and L286V mutations, under the control of the Thy1 promoter [30]. Genotyping was performed according to Jackson Laboratory’s instructions, and was consistent with our previous reports [31]. Animals were sacrificed between 1.2 and 14 months of age (see Supplemental Tables S1 and S2 for details). For age-specific ORI analyses, animals with their hippocampal sections subjected to ORI (n = 21/genotype) were grouped as follows: 2 months (mean 1.83 ± 0.52, range 1.2 to 2.5 months), 3.5 months, 7 months (mean 7.17 ± 0.59, range 6.5–8 months), and 14 months. There was no significant difference in average mouse age between the WT control and 5XFAD model within each age group. Hippocampal sections from 5XFAD mice were subjected to Aβ_42_ assay. All animal procedures were approved by and made in accordance with the guidelines of the Institutional Animal Care and Use Committee (IACUC) Office of Animal Welfare of the University of Pennsylvania, Philadelphia, PA, USA.

#### 2.1.2. Mouse Hippocampal Tissue Preparation

After cervical dislocation without anesthesia, mice were decapitated followed by immediate hippocampus extraction, which was completed within ~1 min. One portion of the right hippocampus (sagittal cut, ~1 mm) was immediately placed in a glass-bottom dish (14 mm diameter of glass, Cellvis, D35-14-1.5-N) filled with 1 mL LCIS^+^ solution (i.e., Live Cell Imaging Solution (Invitrogen™, Thermo Fisher Scientific, Waltham, MA, USA) supplemented with 22 mM glucose) for redox imaging, whereas the remaining portion was immediately frozen and used for Aβ_42_ measurement. The time of dissection was recorded for each specimen. After extraction of all hippocampi for the day (typically 5–7 mice), fresh hippocampal samples in glass-bottom dishes were immediately taken to the imaging site for ORI, which started on average ~35 min after dissection. 

### 2.2. ORI of Fresh Hippocampal Tissue and Data Processing

The cut plane of hippocampal samples was tile-imaged with a wide-field microscope (EVOS FL Auto Imager) in the order they were extracted from the mice. The DAPI and GFP filters (excitation and emission windows, as previously described [32]), a 4 × objective (2343 × 1849 µm^2^ per field of view), and a 14-bit depth mono-camera were employed to acquire the images, which were then photo stitched. The time when imaging started for each specimen was also recorded. Each specimen was imaged multiple times over an approximate 2 h period. To prevent tissue from moving while being imaged, a clean one-cent coin was partially laid on top of the ~1 mm thick tissue section, with the larger portion of the coin resting on the plastic part of the dish bottom to reduce potential weight-induced stress on the tissues. 

To perform a metabolic modulation experiment, small and thin tissue slices (<1 mm, n = 5/genotype) were sliced off from two hippocampal sections in the 2-month-old mouse group after redox imaging acquisitions were performed for these sections. Mitochondrial oxidative phosphorylation uncoupler carbonyl cyanide 4-(trifluoromethoxy)phenylhydrazone (FCCP, 30 µM final concentration) was carefully added to the medium, and the redox signals were acquired before and immediately after FCCP addition. Rotenone and antimycin A (ROT/AA, 10 µM/6 µg/mL, final concentrations) were then added to the dishes, followed by immediate imaging [25,26,33,34]. The interval between each drug administration and imaging was <1 min. All drugs were purchased from Millipore Sigma and reconstituted in DMSO. The stock aliquots were stored in a −80 °C freezer. 

The images were processed and quantified using ImageJ and/or Matlab. A tissue-free area was chosen as the background of the image for the NADH or Fp channels. The region of interest (ROI) was drawn along the tissue area. The net global mean value for NADH or Fp was then calculated by subtracting their respective background. These net mean values were then used to quantify the redox ratio Fp/(NADH+Fp) of a specific tissue specimen. Within each age group, data acquired at different times were corrected to the time when the first data were acquired (~35 min after dissection), using the relationship between redox signals and elapsed time. 

### 2.3. Aβ_42_ Quantification

The frozen portion of the dissected hippocampi from twenty 5XFAD mice were homogenized on ice, and then used for quantification of the soluble human Aβ_42_ by ELISA at room temperature, according to the manufacture’s manual (Thermo Fisher Scientific, Waltham, Massachusetts, USA; Cat. # KHB3441). Briefly, 50 µL aliquots of tissue samples and reference standards (0–1000 pg/mL) were loaded into wells in the supplied 96-well plate, and 50 µL of Hu Aβ_42_ detection antibody solution was added to each well. The mixtures were incubated for 3 h with shaking, then the plate was washed four times with supplied 1 × wash buffer. A total of 100 µL anti-rabbit IgG HRP was added to each well and incubated for 30 min, then the plate was washed four times with 1 × wash buffer. A total of 100 µL stabilized chromogen was added to each well and incubated for 30 min in the dark. A total of 100 µL stop solution was added to each well, and the plate was read at 450 nm absorbance with a microplate reader. The Aβ_42_ levels were calculated by fitting to a standard curve using Microplate Manager (Bio-Rad Laboratories, Inc., Hercules, CA, USA) and were expressed in pg/mL.

### 2.4. Statistical Analysis

GraphPad Prism 9 was used for the statistical analyses. The mean values were compared between the WT and 5XFAD groups, using an unpaired Student’s *t*-test and assuming unequal variance, or using one-way ANOVA with Tukey correction for multiple comparisons. When appropriate, the slopes between the WT and 5XFAD groups were also compared using a linear regression model. *p* < 0.05 was considered statistically significant.

## 3. Results

### 3.1. Confirmation of Redox Signals in Hippocampal Tissue

**Figure 1:**
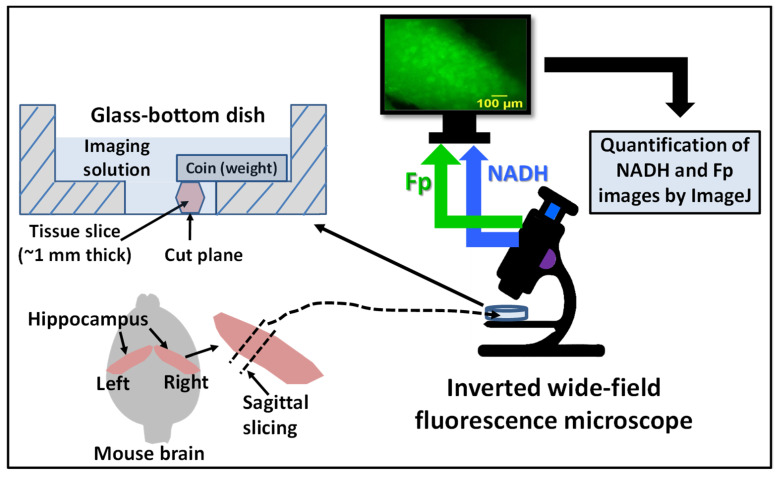
The schematic for ex vivo optical redox imaging of hippocampal slice. Approximately 1 mm thick sagittally cut hippocampal section from a freshly dissected right hippocampus was placed in a 35 mm glass-bottom dish containing imaging solution immediately after slicing. Before imaging, the hippocampal slice was immobilized by a penny coin, with majority of the coin weight resting on the plastic part of the dish bottom. The cut plane was tile-imaged with an inverted wide-field fluorescence microscope equipped with proper optical filters, a 4 × objective lens with a field of view of 2343 × 1849 µm^2^. The raw images of NADH and Fp were processed and quantified using ImageJ, and subsequently underwent further analyses (see Materials and Methods for details).

**Figure 2:**
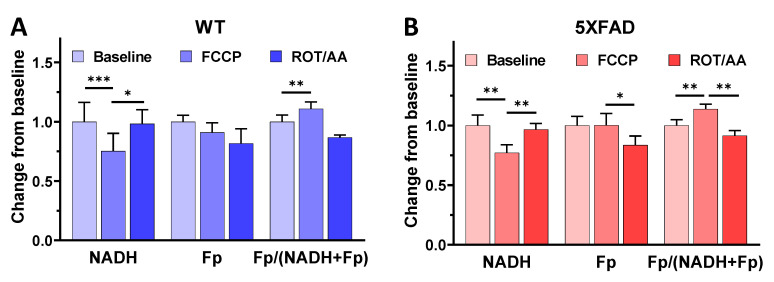
Redox responses of ~1 mm thick hippocampal slices from 2-month-old wild type (**A**) and 5XFAD (**B**) mice to the metabolic modulator FCCP followed by rotenone + antimycin A (ROT/AA). Bars represent normalized mean ± SE (before and after drug administration for the same field of view, n = 5 slices/genotype). *, *p* < 0.05; **, *p* < 0.01; ***, *p* < 0.001 (paired *t*-test).

The schematic for all imaging experiments for this study is shown in Figure 1. We conducted a metabolic modulation experiment to confirm the redox signals and metabolic viability of freshly dissected hippocampal slices [25,33,34]. As expected, after adding mitochondrial oxidative phosphorylation uncoupler FCCP to the culture medium, we immediately observed a ~40% NADH decrease and a ~20% increase in the redox ratio, without a significant change in Fp signals for both the WT and 5XFAD groups (Figure 2). Subsequently, by adding rotenone and antimycin A, which inhibit mitochondrial complex I and III, respectively, we observed an immediate recovery of NADH signal and a decreased redox ratio. These changes indicated that the tissues were viable and confirmed that ORI detected the redox signals in the hippocampal slices.

### 3.2. Redox Kinetics during Imaging in the Hippocampal Tissue Sections

**Figure 3:**
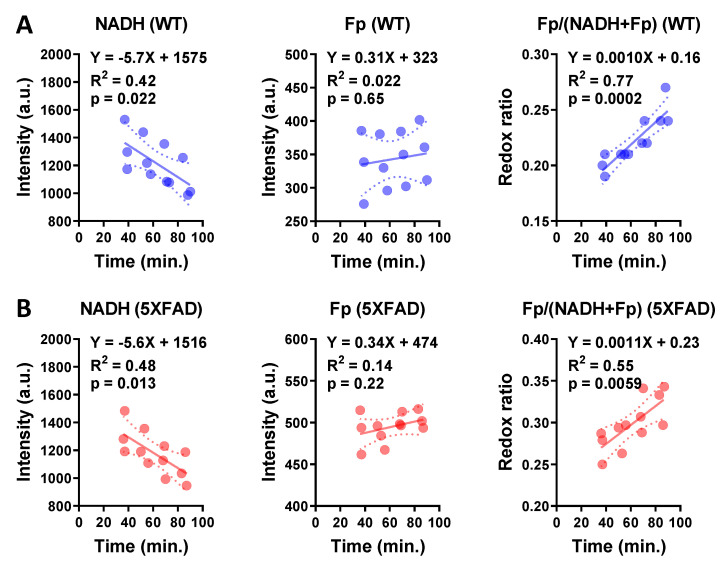
The typical redox changes in cultured hippocampal sections of 6.5-month-old WT (**A**) and 5XFAD mice (**B**). Each dot represents one hippocampal section from an individual mouse and each section was imaged at four time points.

Since there was a time lapse between tissue dissection and imaging, we monitored the redox signals of ex vivo hippocampal tissue sections in the imaging solution (see Materials and Methods for details) for over 1–2 h for each imaging session, then linearly fit the data to obtain the slope and intercept. This allowed us to ensure that all redox data within and across age groups were compared at the same time point. We observed that over time, in both the WT and 5XFAD groups, NADH decreased linearly (*p* = 0.022, 0.013, respectively), while Fp signals essentially stayed the same, and the redox ratio increased linearly (*p* = 0.0002, 0.0059, respectively) with similar slopes (Figure 3).

### 3.3. Relationship between the Redox Indices and Age

**Figure 4:**
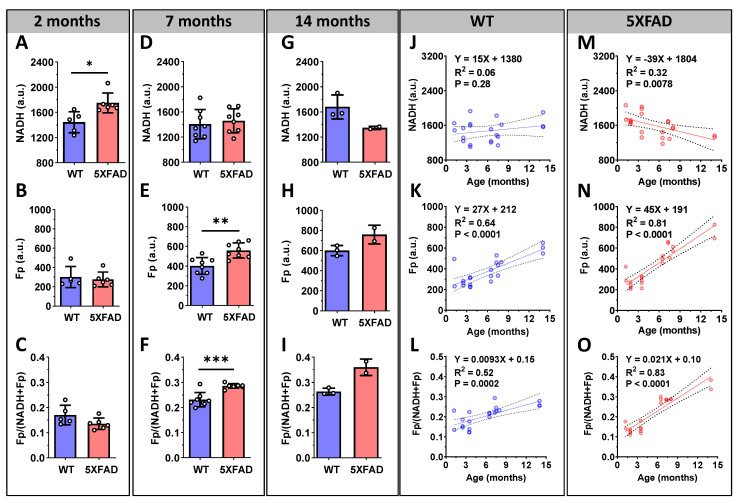
The relationship between redox indices and age in the hippocampi of 5XFAD and wild type mice. Quantified hippocampal redox indices in 2 month (**A**–**C**), 7 month (**D**–**F**), and 14 month (**G**–**I**) groups are graphed with mean ± SD (*, *p* < 0.05; **, *p* < 0.01; ***, *p* < 0.001) and individual mouse values (dots). Linear regressions were performed to represent the trend of time courses of the redox indices (each dot represents the hippocampus from an individual mouse) for WT (**J**–**L**) and 5XFAD (**M**–**O**).

To determine whether and how the redox indices (NADH, Fp, and the redox ratio) changed as mice aged, we quantified the hippocampal redox indices from 21 WT and 21 5XFAD mice aged 1.2 to 14 months (see Supplementary data tables for details). These mice were grouped into four age groups: 2, 3.5, 7, and 14 months. These ages are related to their neuropathological and phenotypical characterizations: amyloid-β accumulation from 1.5–2 months; behavioral and synaptic impairment from 4 months; synaptic and neuronal loss from 7 months onward. The within group analyses revealed a ~21% increase in NADH signal in the hippocampi of 5XFAD mice compared to their WT littermates for the 2 month group (*p* < 0.05, Figure 4A), no significant change in Fp (Figure 4B), and a trend in a more reduced redox ratio (*p* = 0.13, Figure 4C). For the 3.5 mo. group, there was no significant difference in any of the redox indices between the WT and AD hippocampi (data not shown). For the 7 month group, the 5XFAD hippocampi showed a ~41% higher Fp content (*p* = 0.002) and a ~24% higher redox ratio (*p* = 0.0007) relative to the WT, but there were no significant NADH differences (*p* = 0.6) (Figure 4D–F). Only two 5XFAD samples were imaged in the 14 month group (due to an unexpected death of a 5XFAD mouse before this experiment); therefore, no statistical analysis was performed for this group. 

Consistent with these results, the linear regression of NADH, Fp, and the redox ratio with age (Figure 4G–L) showed no significant correlation between NADH and age in the WT hippocampi; however, it did show a significant linear NADH decrease in the 5XFAD hippocampi with advancing age (*p* = 0.0078). Furthermore, although both Fp and the redox ratio increased linearly with age in both the WT control and 5XFAD model, the rate of increase was larger in 5XFAD mice (Figure 4K). Specifically, the 5XFAD hippocampi showed a significantly larger Fp slope than that of the WT hippocampi (45 versus 27 a.u./month, *p* = 0.012). Similarly, the redox ratio slope for the 5XFAD hippocampi was more than two-fold greater than that of the WT mice (*p* < 0.001). We did not observe any difference in the redox indices between male and female mice in either the 5XFAD or WT group.

### 3.4. Correlations between the Redox Indices and Aβ42 Level in the Hippocampi of AD Mice

**Figure 5:**
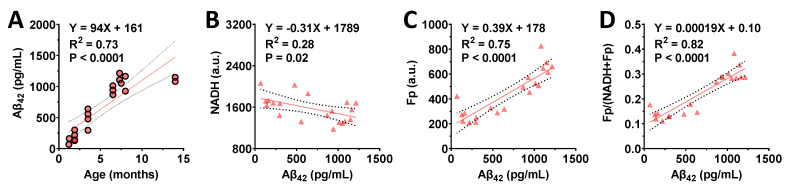
Relationships between Aβ_42_ level (pg/mL) and redox indices in the 5XFAD hippocampi. (**A**) a positive linear correlation between Aβ_42_ level and mouse age; (**B**) a negative linear correlation between NADH and Aβ_42_; (**C**) a positive linear correlation between Fp and Aβ_42_; (**D**) a positive linear correlation between the redox ratio and Aβ_42_.

Using the snap-frozen portions of the hippocampal tissues, we measured human Aβ_42_ levels in the same 5XFAD mice that were subjected to ORI. WT mice do not express human Aβ_42_. We detected human Aβ_42_ accumulation in the 5XFAD hippocampi when mice were as young as 1.5 months. As expected, Aβ_42_ levels increased as mice aged [30] despite the large variations among individuals, and there was a positive linear correlation between Aβ_42_ level and age with high significance (Figure 5A). We further performed linear regression to identify possible correlations between the redox indices and Aβ_42_ level. We found there was a negative linear correlation between Aβ_42_ and NADH (*p* = 0.02; Figure 5B), and a positive linear correlation between Aβ_42_ and Fp (*p* < 0.0001; Figure 5C), as well as Aβ_42_ and the redox ratio *p* < 0.0001; Figure 5D). We did not observe any significant sex difference in either age or Aβ_42_ level in 5XFAD mice.

## 4. Discussion

### 4.1. The Hippocampal ORI Measures Differentiate Normal Aging from AD

We performed optical redox imaging to investigate the NAD redox status in freshly dissected hippocampi from both WT and 5XFAD mice spanning from ~1 to 14 months of age. The 5XFAD model has been demonstrated to recapitulate key pathological features of AD patients, such as progressive amyloid pathology, neuronal loss, and cognitive dysfunction, as well as gene expression patterns and pathway activities similar to human late-onset AD [35]. The 5XFAD model exhibits intraneuronal soluble Aβ_42_ accumulation starting at 1.5 months of age before plaques form, and the appearance of cognitive dysfunction around 4–5 months, when there is extensive amyloid pathology [30,36]. Temporal gene profiling of the 5XFAD neocortex and hippocampus also showed a clear region-specific shift in gene expression patterns between 1 month (pre-symptomatic stage) and 4 months (prodromal stage), with the most dramatic changes occurring from 4 months onwards, including regulatory genes involved in inflammatory and immune processes [37]. In parallel with these established features, we observed significant age-dependent oxidized redox shifts in the hippocampus of both 5XFAD and WT mice over the 2–14 month period, with a larger shift in 5XFAD than in WT mice. 

NAD^+^ deficiency has previously been described in normal brain aging and neurodegenerative disorders, including AD in various models [13]. Owing to the intrinsic fluorescence properties of NADH, aging induced NADH changes have been studied by optical fluorescence methods. For example, an in vitro study measuring NADH and FAD fluorescence intensity in cultured hippocampal and cortical neurons isolated from 3xTg-AD and non-transgenic control mice at various ages showed that NADH intensity continuously increased between 2 to 11 months, with levels significantly lower in the 3xTg-AD versus the non-transgenic control neurons from 2 months onwards [9]. The same study also showed that the redox ratio NADH/FAD followed the same trend as that of NADH in non-transgenic control neurons between 2–21 months, whereas this ratio continued to decline from 2 months onwards in 3xTg-AD neurons. In our study, we found that the hippocampal NADH level was significantly higher in 2 month 5XFAD mice than in age-matched WT littermates. While we did not observe a significant NADH change with age in WT hippocampal tissue, the hippocampal NADH linearly decreased with age in 5XFAD mice. Furthermore, we found a larger age-dependent increase in the redox ratio Fp/(NADH+Fp), i.e., a shift to a more oxidized state due to both decreasing NADH and increasing Fp in the 5XFAD hippocampi, consistent with the aforementioned report on neurons isolated from 3xTg-AD mice, showing a continuous decrease in the NADH/FAD ratio with age [9]. Note that their redox ratio defined as NADH/FAD is inversely related to our definition Fp/(NADH+Fp). Another study of isolated mouse hippocampal neurons, which employed a fluorescence lifetime imaging microscopy technique, demonstrated that it was the free NADH that significantly decreased due to normal aging and, more so, due to AD (3xTg-AD mouse); additionally, there was also a significant shift from free to protein-bound NADH [14]. However, there are no studies to date reporting that NADH changes at the tissue level in the mouse brain of AD models by any imaging modality.

### 4.2. The ORI Measures Correlate with Hippocampal Aβ_42_ Level in 5XFAD Mice

Since Aβ level in the 5XFAD model indicates AD progression, the significant associations between the redox indices and Aβ_42_ levels we observed support the notion that hippocampal NAD redox status is indicative of AD pathology. Oxidative stress is associated with damaged Aβ peptide and contributes to AD progression [38]. Furthermore, oxidative stress was shown to be one of the biological processes associated with differentially expressed proteins in 5XFAD and WT mice at the age of 4 months [39]. We previously reported that a higher redox ratio correlates with higher reactive oxygen species (ROS), generated by oxidative insults [25,26,28], and higher oxidative stress in inflamed rhesus macaques brains [29]. As NAD redox abnormality occurs earlier than ROS-induced oxidative stress on macromolecules and is an upstream event in isolated neurons from AD mice of various ages [9,15], it suggests that our observation that a decreased NADH, an increased Fp, and a more oxidized redox status correlated with the increase of Aβ in the 5XFAD hippocampi is consistent with the understanding that oxidative stress underlies AD development in 5XFAD mice. It remains to be tested in the future whether the alterations of ORI measures in the early stage can predict the disease progression and severity in the late stages of AD.

### 4.3. Limitations of Our Study and Open Questions

There are several technical limitations in the study. First, we took a low-resolution imaging approach with a wide-field fluorescence microscope. This low-cost and convenient approach is sufficient for global averaging analysis to differentiate between AD and WT models, but it does not provide finer details regarding the redox distribution within tissue. An alternative approach is to employ an upright two-photon microscope that provides much higher resolution for imaging ex vivo tissue in investigating regional and cellular redox changes within brain tissues, as we observed in freshly dissected mouse leg muscles [32]. Second, as all tissue glues tested have strong fluorescence in the NADH channel, we had to alternatively place a coin to immobilize the tissue section. It is unclear whether this could put the tissue under significant mechanical stress, despite most of the coin weight being placed on the plastic portion of the dish bottom (Figure 1). However, since both WT and 5XFAD tissues were imaged the same way, such mechanical stress should not differentially affect their metabolism. Third, ORI cannot differentiate between NADH and NADPH signals; therefore, the NADH measures reported here include the contributions by both fluorophores, but NADPH signal is much weaker in brain tissues [40]. 

In addition, the optically determined redox ratio may or may not reflect the NAD^+^/NADH redox potential measured by other means. The optical redox ratio FAD/(NADH+FAD) was demonstrated to be linearly proportional to NAD^+^/(NADH+NAD^+^), determined by liquid chromatography coupled with mass spectroscopy (LC/MS-MS) in differentiating stem cell cultures [41] or precancerous tissues [42]. In contrast, breast cancer cells that have a compromised NAD^+^ synthesis demonstrated a reductive shift in NAD^+^/NADH ratio, as measured by biochemical assay [43], but an oxidative shift in the optical redox ratio [27]. Therefore, it is likely that, under certain conditions, the positive linear correlation between NAD^+^/(NADH+NAD^+^) and the optical redox ratio may not hold. In this study, we found that the optical redox ratio increased with age and, more so, with AD progression in the hippocampal tissues. In comparison, the NAD^+^/NADH ratio measured in vivo by phosphorous-31 magnetic resonance spectroscopy in the occipital lobe of normal human brains were reported to decrease with age [44]. This apparent discrepancy in the age trend for redox ratios might be interpreted by the fact that the optical redox ratio has two redox sensors: the NADH and FAD. Thus, the optical redox ratio Fp/(NADH+Fp) is a parameter that can be affected by both the bioenergetics and oxidative stress. With aging, NAD^+^/NADH may decrease, reflecting lower bioenergetics, whereas the optical redox ratio may increase due to increased oxidative stress. Nevertheless, more investigations are needed to further understand the biological basis of the optical redox ratio. 

Lastly, the primary goal of this study was to explore whether optical redox imaging can detect aging effects and reveal metabolic differences between normal aging and AD-induced metabolic abnormality. Potential sex differences were not part of the original study design, but we tested it during data processing. Studies of sex difference with this a priori hypothesis should be considered in the future with a more balanced research design, including even animal numbers. In addition, subsequent studies should explore potential changes in NADH, Fp, and the redox ratio in other brain regions, such as the cortex.

## 5. Conclusions

Employing a wide-field fluorescence microscope, this study examined the utility of ex vivo optical redox imaging of freshly dissected hippocampal tissue sections from 5XFAD and WT mice to study Alzheimer’s disease in comparison with normal aging. The ORI method reported here detected significant redox differences between the AD and normal hippocampi. To the best of our knowledge, our study provides the first imaging measurements of NADH, Fp, and the redox ratio at the tissue level of AD models at various ages. Our findings suggest that the ORI can be further optimized to conveniently study the metabolism of freshly dissected brain tissues in animal models. Furthermore, NADH abnormality could be an early event of AD; thus, further investigation of its role in disease pathology and its potential as an early biomarker for AD is warranted.

## Data Availability

Data is contained within the article or supplementary material.

## Supplementary Materials

The following supporting information can be downloaded at: https://www.mdpi.com/article/10.3390/metabo12090786/s1, Table S1: Experimental data (WT hippocampus); Table S2: Experimental data (5XFAD hippocampus).
