# Supplementary material for: Optical Redox Imaging of Ex Vivo Hippocampal Tissue Reveals Age-Dependent Alterations in the 5XFAD Mouse Model of Alzheimer’s Disease
Source: Metabolites. 2022 Aug 25;12(9):786. doi: 10.3390/metabo12090786 (PMC9504813; doi:10.3390/metabo12090786)
Supplement: Supplementary file 1 [file metabolites-12-00786-s001.zip › metabolites-1862317-supplementary.pdf]

metabolites MDPI

Optical Redox Imaging of Ex Vivo Hippocampal Tissue Reveals Age-Dependent Alterations in the 5XFAD Mouse Model of Alzheimer's Disease

Table S1. Experimental data (WT). NA: not measured.

Age (month)	Age Group (month)	Genotype	Sex	AB_{42} (pg/mL)	NADH (a.u.)	Fp (a.u.)	Fp/(NADH+Fp)
1.2	2	WT	F	NA	1648	493	0.231
1.25	2	WT	F	NA	1485	231	0.134
2.5	2	WT	F	NA	1313	238	0.153
2.5	2	WT	F	NA	1538	263	0.146
2.5	2	WT	F	NA	1238	283	0.186
3.5	3.5	WT	M	NA	1143	246	0.177
3.5	3.5	WT	F	NA	1104	318	0.224
3.5	3.5	WT	M	NA	1927	309	0.139
3.5	3.5	WT	F	NA	1594	222	0.123
3.5	3.5	WT	F	NA	1628	226	0.122
6.5	7.5	WT	F	NA	1208	276	0.199
6.5	7.5	WT	F	NA	1231	330	0.219
6.5	7.5	WT	F	NA	1511	390	0.215
8	7.5	WT	F	NA	1615	463	0.232
7.3	7.5	WT	M	NA	1373	529	0.294
7.3	7.5	WT	M	NA	1825	459	0.221
7.5	7.5	WT	M	NA	1338	435	0.245
7.5	7.5	WT	M	NA	1138	334	0.227
14	14	WT	M	NA	1901	649	0.255
14	14	WT	F	NA	1561	604	0.279
14	14	WT	F	NA	1579	547	0.257

Table S2. Experimental data (5XFAD). NA: not measured.

Age (month)	Age Group (month)	Genotype	Sex	AB_{42} (pg/mL)	NADH (a.u.)	Fp (a.u.)	Fp/(NADH+Fp)
1.2	2	5XFAD	F	68	2065	422	0.176
1.25	2	5XFAD	F	162	1732	286	0.142
1.9	2	5XFAD	M	129	1718	273	0.137
1.9	2	5XFAD	M	141	1637	221	0.119
1.9	2	5XFAD	F	300	1671	248	0.129
1.9	2	5XFAD	F	219	1682	209	0.110
3.5	3.5	5XFAD	M	NA	1986	266	0.118
3.5	3.5	5XFAD	F	638	1865	318	0.146
3.5	3.5	5XFAD	F	478	2031	327	0.139
3.5	3.5	5XFAD	M	295	1443	214	0.129
3.5	3.5	5XFAD	F	557	1320	288	0.179
8	7.5	5XFAD	M	927	1524	572	0.289
8	7.5	5XFAD	F	1165	1552	613	0.288
7.3	7.5	5XFAD	F	1107	1694	648	0.284
7.3	7.5	5XFAD	F	1214	1684	661	0.287

7.5	7.5	5XFAD	F	1053	1288	508	0.283
6.5	7.5	5XFAD	M	942	1444	494	0.269
6.5	7.5	5XFAD	M	868	1308	523	0.302
6.5	7.5	5XFAD	F	1013	1176	454	0.288
14	14	5XFAD	F	1148	1364	693	0.337
14	14	5XFAD	F	1082	1329	826	0.383
